# Derivation and validation of a predictive model for subtotal cholecystectomy

**DOI:** 10.1007/s00464-024-11241-8

**Published:** 2024-09-16

**Authors:** James Lucocq, David Hamilton, Abdelwakeel Bakhiet, Fabiha Tasnim, Jubayer Rahman, John Scollay, Pradeep Patil

**Affiliations:** Department of General and Upper GI Surgery, Ninewells Hospital, Dundee, UK

**Keywords:** Cholecystectomy, Laparoscopy, Subtotal cholecystectomy, Prediction, Acute cholecystitis, Complications, Derivation

## Abstract

**Introduction:**

Rates of subtotal cholecystectomy (STC) are increasing in response to challenging cases of laparoscopic cholecystectomy (LC) to avoid bile duct injury, yet are associated with significant morbidity. The present study identifies risk factors for STC and both derives and validates a risk model for STC.

**Methods:**

LC performed for all biliary pathology across three general surgical units were included (2015–2020). Clinicopathological, intraoperative and post-operative details were reported. Backward stepwise multivariable regression was performed to derive the most parsimonious predictive model for STC. Bootstrapping was performed for internal validation and patients were categorised into risk groups.

**Results:**

Overall, 2768 patients underwent LC (median age, 53 years; median ASA, 2; median BMI, 29.7 kg/m^2^), including 99 cases (3.6%) of STC. Post-operatively following STC, there were bile leaks in 29.3%, collections in 19.2% and retained stones in 10.1% of patients. Post-operative intervention was performed in 29.3%, including ERCP (22.2%), laparoscopy (5.0%) and laparotomy (3.0%). The following variables were positive predictors of STC and were included in the final model: age >  60 years, male sex, diabetes mellitus, acute cholecystitis (AC), increased severity of AC (CRP > 90 mg/L), ≥ 3 biliary admissions, pre-operative ERCP with/without stent, pre-operative cholecystostomy and emergency LC (AUC = 0.84). Low, medium and high-risk groups had a STC rate of 0.8%, 3.9% and 24.5%, respectively.

**Discussion:**

The present study determines the morbidity of STC and identifies high-risk features associated with STC. A risk model for STC is derived and internally validated to help surgeons identify high-risk patients and both improve pre-operative decision-making and patient counselling.

Salvage techniques (e.g. subtotal cholecystectomy [STC], fundus-first cholecystectomy) have been utilised in challenging cases of laparoscopic cholecystectomy (LC) to minimise the risk of bile duct injury (BDI) [1–3]. Rates of subtotal cholecystectomy (STC) are reportedly increasing with a concomitant decline in the rates of conversion to open in difficult cholecystectomy [4, 5]. Although STC is performed to reduce the risk of BDI, the procedure can be complicated by a myriad of other concerning complications (e.g. recurrent cholecystitis, bile leak, retained stones). A previous study by our study group demonstrated the significant relative morbidity of subtotal laparoscopic cholecystectomy compared to conventional laparoscopic cholecystectomy, including higher rates of bile leak, retained stones and post-operative intervention [6].

Given the disparate peri-operative course in STC, clinicians should aim to identify high-risk patients for STC pre-operatively. This is particularly relevant in poor surgical candidates, who may not be able to tolerate the repercussions of such complications.

Previous studies have identified several risk factors for STC including acute cholecystitis (AC), endoscopic retrograde cholangiopancreatography (ERCP), the number of previous admissions and cholecystostomy [6, 7]. To achieve adequate risk stratification for informed patient consent and peri-operative decision-making, these risk factors should be amalgamated to form an accurate pre-operative assessment. The aim of the present study was to identify risk factors for STC and both derive and validate a risk model for STC.

## Methods

LC performed for all biliary pathology across three general surgical units between 2015 and 2020 were included. One unit was a tertiary-care unit and the other two were satellite units for elective LC. The surgical units were located in a defined geographical region with a population of approximately 490,000 people.

In our unit the routine approach to managing suspected choledocholithiasis was magnetic resonance cholangiopancreatography (MRCP) and pre-operative ERCP. Planned common-bile duct exploration was not routinely practiced [8–10]. Furthermore, the standard approach of LC by all general surgeons was to establish the critical view of safety [11, 12]. Ethical approval was granted by the regional information governance committee.

### Definition of STC and CC

All STC and conventional cholecystectomy (CC) were identified retrospectively from multiple regional databases using a deterministic records-linkage methodology. In each case of cholecystectomy the operative approach was at the discretion of each surgeon but generally each surgeon followed the ‘ABCD of safe laparoscopic cholecystectomy’ described by Gupta. This aimed to achieve a conventional cholecystectomy by achieving the critical view of safety before clipping the cystic duct and artery [13]. STC were confirmed using the written operation notes and operative details were noted. In the present study a STC was defined as any cholecystectomy where a remnant Hartmann’s pouch was left in-situ. Previous systematic reviews have highlighted the heterogeneity of operative technique in STC, including cases defined solely by the posterior wall being left in-situ [5, 14]. In the present study, such a definition, where the posterior wall is retained but Hartmann’s pouch is removed, was not considered STC. Leaving the posterior wall is considered a safe approach in difficult cholecystectomy to avoid liver injury and haemorrhage but has little clinical implication [15].

Although the previous study by our study group investigated the relative morbidity of subtotal laparoscopic cholecystectomy versus conventional laparoscopic cholecystectomy, the present study included all subtotal and conventional cholecystectomy, including those that required open conversion. These patients were included to minimise bias when aiming to create a predictive model.

### Data collection

Clinicopathological features were collected and included demographics, American society of anaesthesiology score (ASA), BMI, comorbidities (myocardial infarction [MI], cerebrovascular accident [CVA], chronic obstructive pulmonary disease [COPD] and diabetes mellitus [DM]), previous abdominal surgery, previous upper abdominal surgery, number of admissions, indication for cholecystectomy, severity of AC, pre-operative ERCP ± stent insertion, pre-operative cholecystectomy and timing of cholecystectomy.

### Data analysis

Intra-operative details were reported including length of procedure, complications, intra-operative drain placement, and the rate of intra-operative cholangiogram. Patients were followed up for 100 days for post-operative outcomes. These included complication rates, pathology findings, post-operative length of stay (LoS), readmission and mortality. Prolonged LoS was defined as > 2 days as previously defined in other studies [16, 17].

Following this, a model was derived and validated for the prediction of STC using R Studio version 2024.04.2. All variables were included initially and using backward step multivariate logistic regression, insignificant variables were removed in stepwise fashion to derive the most parsimonious model. Variance inflation factor (VIF) was used to assess multicollinearity between variables and a value greater than 5 indicated significant collinearity. In cases of multicollinearity, variable selection for elimination was guided by previous literature and by the relationship between each variable and STC. The severity of AC was defined by the inflammatory markers on the most recent admission or assessment prior to cholecystectomy. Admission c-reactive protein (CRP) was split into quartiles and severe cholecystitis was defined as the highest CRP quartile (> 90 ml/L). Age was grouped as follows: age < 40, 40–60 and > 60 years.

With the aim of creating a simple model, final variables were allocated a score of 1. The cumulative score for each patient was calculated and the discriminative ability of the model was assessed using a ROC curve. The model was validated using 1000 bootstrap samples and the concordance index (C-index) of a test cohort was reported. Calibration was then assessed by the model slope and illustrated with a calibration plot.

To determine the levels of contribution from each factor the ‘Variable Importance’ function within the ‘randomForrest’ machine learning algorithm was used. The mean decrease accuracy (%incMSE) and the mean decrease Gini (IncNodePurity) were reported. The mean decrease accuracy reports the decrease in accuracy by omitting individual variables. The mean decrease Gini uses the Gini impurity index to determine what degree each variable contributes to the homogeneity of the nodes in the model.

The risk of STC was then reported for each cumulative risk score and risk scores were categorised into low, medium and high-risk groups.

## Results

Overall, 2768 patients underwent LC (median age, 53 years; median ASA, 2; median BMI, 29.7 kg/m^2^) and included a total of 602 emergency LC (21.7%) and 2166 elective LC (78.3%) (ratio 1:3.5). The incidence was 133 LC per 100,000.

There were 99 STC (3.6%) in the overall cohort, including 91 cases of laparoscopic STC and 8 cases of conversion to open STC. The reasons for STC were as follows: significant adhesions to the cholecystohepatic triangle inhibiting safe dissection (67 cases; 67.7%) associated with impacted stones in 7 cases and an intrahepatic gallbladder in 4 cases; cholecystoduodenal fistula/adhesions (9 cases, 9.1%); significant adhesions from Hartmann’s pouch to the common bile duct (7 cases; 7.1%); gallbladder mass (4 cases; 4.0%); cholecystocolic fistula/adhesions (3 cases; 3.0%); Mirizzis (3 cases; 3.0%); unclear ductal anatomy (3 cases; 3.0%) and a shrunken/retracted gallbladder (3cases; 3.0%) one of which followed a cholecystostomy. Additional findings included empyema in (22 cases, 22.2%), gangrenous gallbladder (22 cases, 22.2%) and gallbladder perforation (11 cases, 11.1%).

The reasons for conversion to open in the 8 cases were cholecystohepatic adhesions rendering further dissection unsafe (4 cases), significant adhesions from Hartmann’s pouch to the CBD (1 case), cholecystocolic adhesions resulting in a colonic injury (1 case), cholecystoduodenal adhesions resulting in a duodenal injury (1 case) and a large left liver lobe and obscuring transverse colon in another case.

In 76 cases (76.7%) a fenestrated STC was performed, in 10 cases (10.1%) a reconstituting STC and in the remaining cases the status of Hartmann’s pouch was unclear. In 64 cases (64.6%) the posterior wall was left in-situ.

### Risk factors for STC

Table 1. compares the clinicopathological features of STC and CC and identifies risk factors for STC. Identified risk factors for STC were the following: higher age (*p* < 0.001), male sex (*p* < 0.001), DM (*p* < 0.001), AC (*p* < 0.001), choledocholithiasis (*p* < 0.001), higher CRP (*p* < 0.001), higher number of pre-operative admissions (*p* < 0.001), pre-operative ERCP (*p* = 0.001) and pre-operative cholecystostomy (*p* < 0.001).

**Table 1:** Pre-operative characteristics of STC and CC

Variable (%)	STC (*n* = 99)	CC (*n* = 2669)	*p*-value
Median age	62	53	< 0.001
Male Sex	55 (55.6)	694 (26.0)	< 0.001
Median BMI	30	29.5	0.546
Median ASA	2	2	0.324
Previous abdominal surgery	23 (23.2)	540 (20.2)	0.467
Previous Upper Gastrointestinal Surgery	4 (4.0)	44 (1.6)	0.073
Comorbidities			
MI	4 (4.0)	80 (3.0)	0.552
CVA	4 (4.0)	65 (2.4)	0.314
COPD	5 (5.1)	63 (2.4)	0.090
DM	14 (14.1)	143 (5.4)	< 0.001
Number of emergency admissions			
1	58 (58.6)	1149 (43.4)	0.002
2	20 (20.2)	218 (8.2)	< 0.001
≥ 3	12 (12.1)	57 (2.2)	< 0.001
Indication*			
Acute cholecystitis	79 (79.8)	896 (33.6)	< 0.001
Choledocholithiasis	24 (24.2)	248 (9.3)	< 0.001
Gallstone pancreatitis	15 (15.1)	279 (10.5)	0.136
Biliary colic	11 (11.1)	1469 (55.0)	< 0.001
Admission CRP			
CRP20-90	21 (21.2)	187 (7.0)	< 0.001
CRP ≥ 90	30 (30.3)	179 (6.7)	< 0.001
Pre-operative ERCP	31 (31.3)	247 (9.3)	< 0.001
With stent	6 (6.0)	35 (1.3)	0.001
Pre-operative Cholecystostomy	8 (8.1)	32 (1.2)	< 0.001

*Mutually inclusive

### Outcomes of STC

The median operative time for STC was 125 min (Table 2). In 38.4% of STC the procedure was performed as an emergency, compared with 21.1% of CC (*p* < 0.001). Intraoperatively there were 5 cases (5.1%) of bowel injury and 5 cases (5.1%) of intra-operative bile leak in the STC cohort. Twenty nine patients (29.2%) had blood loss 100–500mls and five patients (5.0%) had >500mls blood loss. No bile duct injuries occurred in the STC cohort.

**Table 2:** Operative details of STC and CC

Variable	STC (*n* = 99)	CC (*n* = 2669)	*p*-value
Median operation time (min)	125	73	< 0.001
Emergency vs. elective	38 (38.4)	564 (21.1)	< 0.001
Conversion to open	8 (8.1)	23 (0.9)	< 0.001
Intra-operative cholangiogram	8 (8.1)	35 (1.3)	< 0.001
Intra-operative drain	90 (90.9)	125 (4.7)	< 0.001
Intra-operative complication			
Bowel injury	5 (5.1)	4 (0.1)	< 0.001
Bile leak	5 (5.1)	17 (0.6)	< 0.001
Significant haemorrhage	5 (5.1)	14 (0.5)	< 0.001
Bile duct injury	0 (0.0)	3 (0.1)	0.738

Post-operatively, there were bile leaks in 29.3%, collections in 19.2% and retained stones in 10.1% of the STC cohort (Table 3). Post-operative interventions were performed in 29.3% which included ERCP (22.2%), laparoscopy (5.0%) and laparotomy (3.0%). There were two cases of gallbladder cancer in the STC group, both of which presented as a gallbladder mass at laparoscopy. Xanthgranulomatous cholecystitis was more common in the STC group compared to the CC group (7.1 vs. 0.8%; *p* < 0.001).

**Table 3:** Post-operative outcomes

Variable	STC (*n* = 99)	CC (*n* = 2669)	*p*-value
Post-operative complication			
Retained stone	10 (10.1)	35 (1.3)	< 0.001
Collection	19 (19.2)	36 (1.3)	< 0.001
Bile leak	29 (29.3)	28 (1.0)	< 0.001
Post-operative intervention	29 (29.3)	65 (2.4)	< 0.001
ERCP	22 (22.2)	44 (1.6)	< 0.001
Laparoscopy	5 (5.0)	13 (0.5)	< 0.001
Laparotomy	3 (3.0)	6 (0.2)	< 0.001
Prolonged LoS (> 2 days)	84 (84.8)	213 (8.0)	< 0.001
Readmission	29 (29.3)	183 (6.9)	< 0.001
Pathology			
Gallbladder cancer	2 (2.0)	8 (0.3)	0.005
Xanthogranulomatous cholecystitis	7 (7.1)	21 (0.8)	< 0.001
Mortality	0 (0.0)	3 (0.1)	0.738

Fenestrated STC had higher rates of bile leak compared to reconstituting STC (32.9 vs 0%; *p* = 0.032). The median length of drain insertion was higher in fenestrated compared to reconstituting STC (8 vs. 4 days, *p* = 0.009). There was no significant difference in the re-intervention rate between both groups (31.6 vs. 10.0% *p* = 0.158). The length of stay was significantly higher in fenestrated compared to reconstituting STC (median, 7 vs. 4.5 days, *p* = 0.039. Comparisons of other outcomes between fenestrated and reconstituting STC were limited due to the small number of reconstituting STC.

Of the patients that suffered a bile leak, all 29 patients had intra-operative drains placed. 18 patients (62.1%) underwent an ERCP of which 16 patients received a stent and the remaining two patients had a sphincterotomy. ERCP was performed a median of 8 days after LC (IQR 5–12 days). Of the 29 patients, 2 patients underwent a laparoscopic washout and 2 patients a laparotomy and washout.

### Model derivation

Using backward elimination the most parsimonious model was derived (Table 4). The following variables were included in the final model: age > 60 years, male sex, DM, AC diagnosis, severe AC, ≥ 3 biliary admissions, pre-operative ERCP with/without stent, pre-operative cholecystostomy and emergency LC.

**Table 4:** Most parsimonious model reporting variables associated with STC

Variable	Odds ratio	SE	*P*-value
Age > 60	1.49	0.23	0.034
Male Sex	2.14	0.22	< 0.001
Diabetes Mellitus	1.84	0.33	0.022
Diagnosis			
AC	3.78	0.28	< 0.001
Severe AC (CRP ≥ 91)	2.20	0.26	0.003
≥ 3 biliary admissions	2.46	0.39	0.021
Pre-operative ERCP			
Without stent	2.75	0.27	0.002
With stent	2.83	0.53	0.049
Pre-operative Cholecystostomy	2.14	0.46	0.045
Emergency LC	2.44	0.23	0.001

The was significant multicollinearity (VIF > 5) between choledocholithiasis and ERCP. Since ERCP had a stronger association with STC and previous literature had identified the relationship between ERCP and difficult cholecystectomy, ERCP was retained and choledocholithiasis was removed [18].

### Model validation

**Fig. 1:**
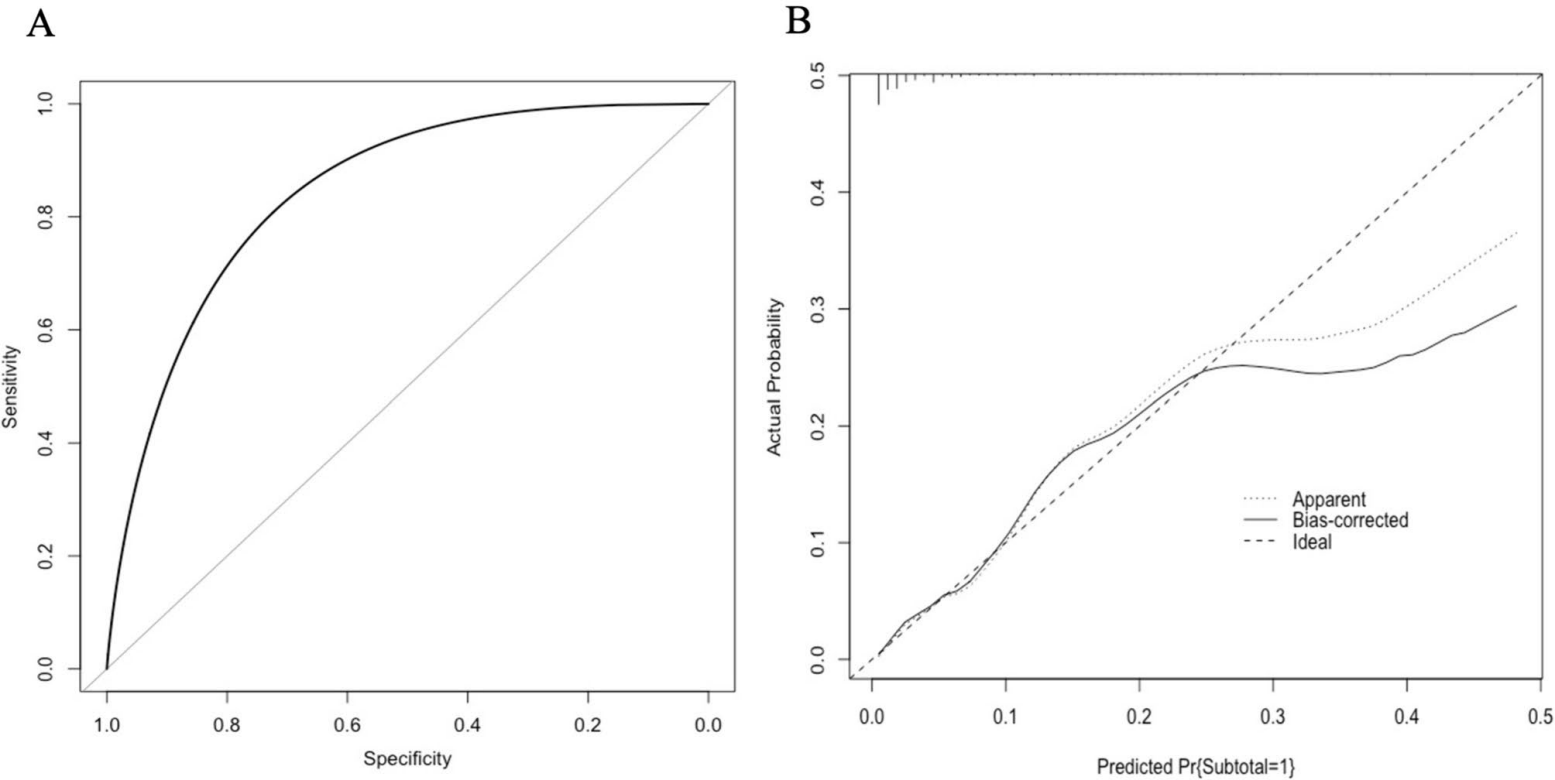
ROC curve (**A**; AUC = 0.84) and calibration plot (**B**) for the STC risk model

**Fig. 2:**
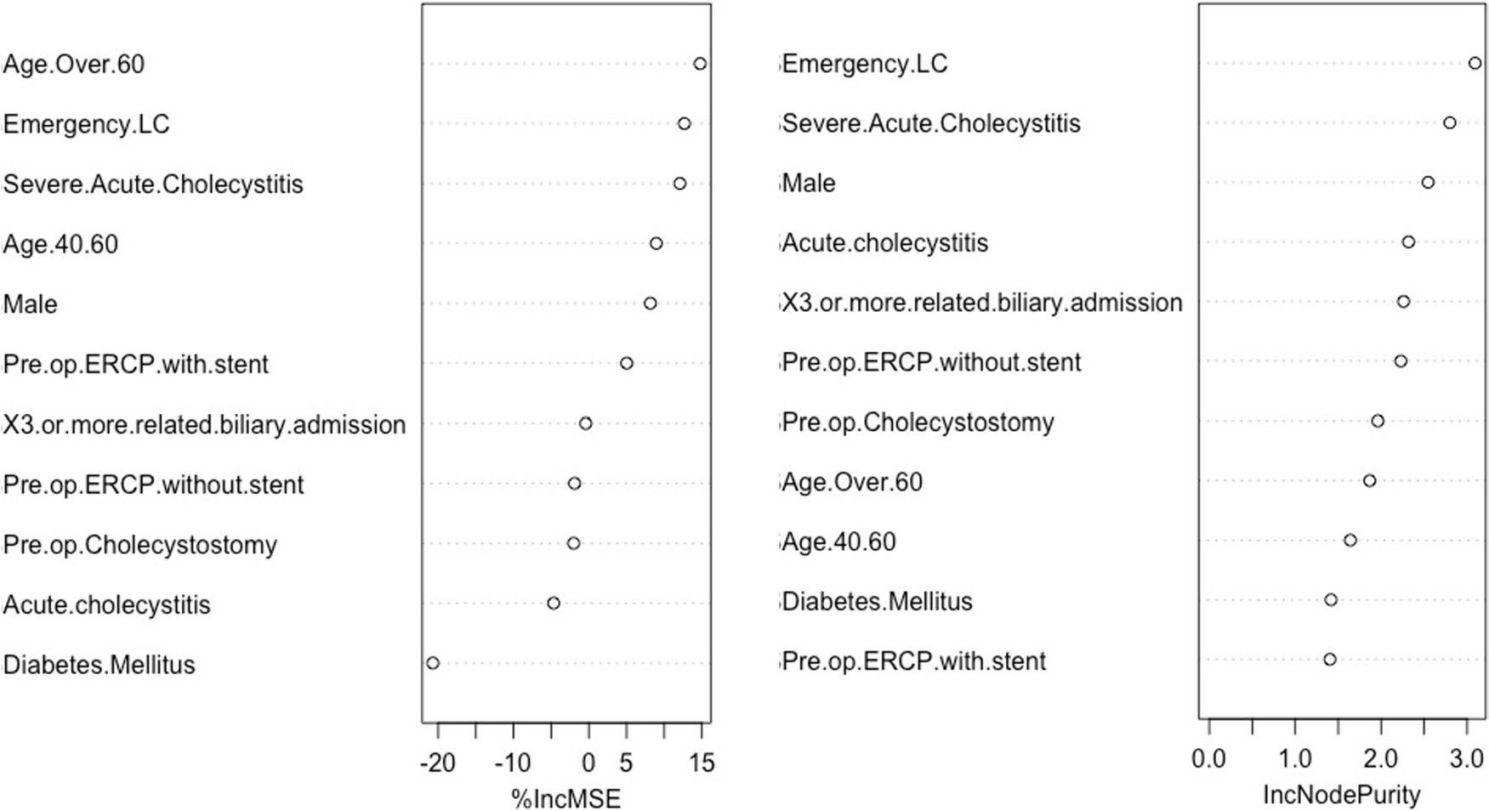
Variable importance for the STC risk model

A ROC curve was plotted to determine the discriminative ability of the model (AUC = 0.84; Fig. 1a). The calibration of the model is also reported below (Fig. 1b) which demonstrated good calibration for predicted probabilities up to 30%. Variable importance was reported using mean decrease accuracy (%incMSE) and the mean decrease Gini (IncNodePurity) (Fig. 2). The most important variables as determined using mean decrease accuracy were higher age (> 60 years), emergency LC and severe AC (CRP > 90 mg/L). The most important variables determined using mean decrease Gini were emergency LC, severe AC and male sex.

**Fig. 3:**
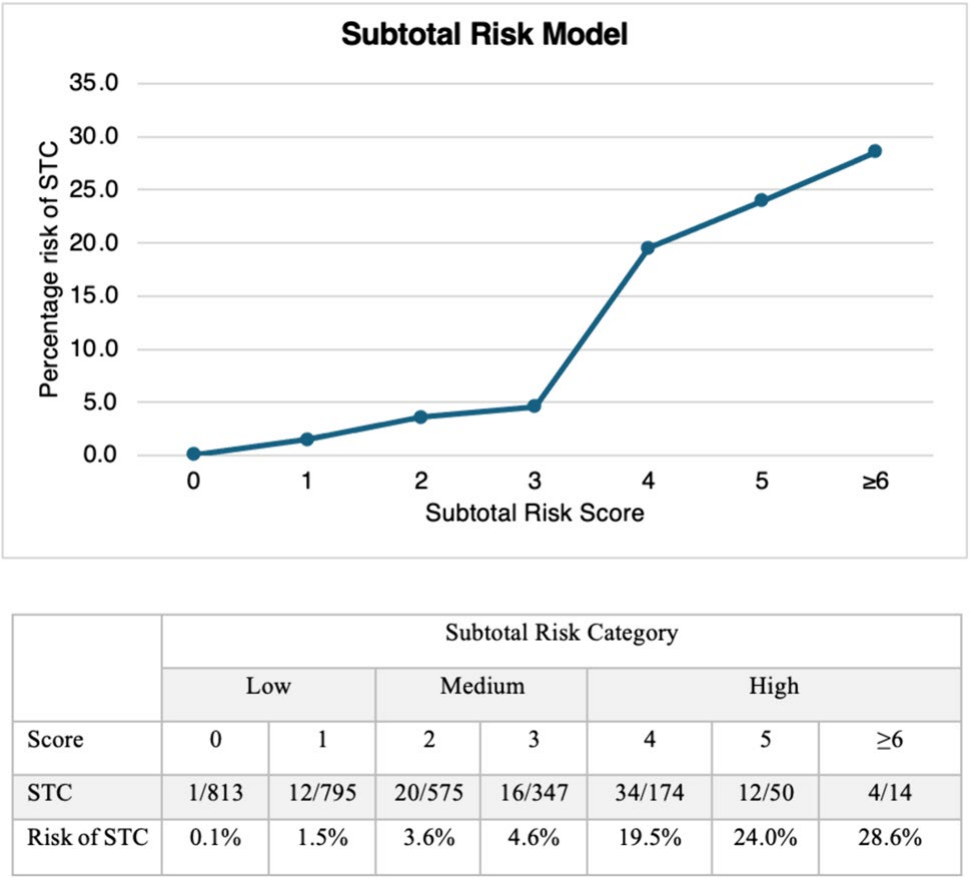
Risk of STC for cumulative risk score calculated from the STC risk model

The risk of STC was plotted for cumulative risk scores (Fig. 3) and risk of STC ranged from 0.1% (Score 1) to 28.6% (Score ≥ 6). Patients were grouped into low (score 0/1), medium (score 2/3) and high-risk (score ≥ 4) groups. Low, medium and high-risk groups had a rate of STC of 0.8%, 3.9% and 24.5%, respectively.

## Discussion

The present study identifies risk factors of STC in a large patient cohort and demonstrates the significant morbidity of STC compared to CC. The paper derives and validates a risk score to help predict the risk of STC and in turn difficult cholecystectomy. Several risk factors are identified, many of which are incorporated into the final risk model and these include demographic, clinical, biochemical and procedural variables.

Systematic reviews by both Henneman et al. and Elshaer et al. have investigated the morbidity associated with STC [5, 14]. Although Elshaer et al. report comparable morbidity rates between STC and CC, both reviews describe the variation in operative technique between studies. In fact, these reviews include cases termed as STC despite cystic duct control being achieved and only the posterior wall being left in-situ [4, 19–21]. In such cases, similar rates of bile leak, collection and retained stones would be expected compared to CC. The present study only includes cases where the cystic duct was not controlled and Hartmann’s pouch was left in-situ, the majority of which were fenestrated STC [22]. The elevated morbidity rates associated with this operative outcome are reported and therefore the morbidity of STC should not be underestimated or deemed comparable with conventional cholecystectomy. Going forward, globally accepted definitions of STC subtypes are required to ensure consistent reporting of outcomes.

One of the biggest risks following STC is a bile leak and the subsequent risk of ERCP [2]. The significant bile leak rate in this study (29.3%) can be explained by the proclivity towards a fenestrated STC and the higher rate of bile leak in fenestrated vs. reconstituting subtypes (*p* = 0.032). However, a reconstituting STC is not without risk; recurrent cholecystitis, further stone formation and biloma can occur following this subtype [22]. Moreover, Lidsky et al. report a concerning rate of bile leak (76.9%) and post-STC ERCP (30.8%) following reconstituting STC, in addition to a completion cholecystectomy rate of 6.2% (4/65) [2].

In the present study, post-STC bile leak was managed with an ERCP in 62% of cases, a median of 8 days following LC (IQR, 5–12). In the remaining patients, the bile leak had dried-up successfully without ERCP with the use of intra-operative drains in all cases. This suggests that ERCP can be avoided in a significant proportion of cases, if delayed-ERCP is adopted. The utilisation of intra-operative drains is also imperative to reduce the re-intervention rate. In patients with an unanticipated bile leak where an intra-operative drain is not placed, Wilson et al. report a re-laparoscopy rate of 97.6% (40/41), which is substantially higher than the present study (13.9%) [23].

Risk factors for STC are outlined, many of which have been identified previously. Farrugia et al. found that ERCP and AC increased the likelihood of STC [7]. In the previous study by our research group, higher age, male sex, cholecystostomy, previous admissions and emergency LC were also identified, many of which are associated with other metrics of difficult cholecystectomy [6, 24–28]. The present study reports diabetes mellitus and the severity of AC as other risk factors but most importantly highlights the cumulative risk of certain risk factors. Since pre-operative ERCP was standard practice for the management of choledocholithiasis and there was inherent multicollinearity with choledocholithiasis, the independent risk of choledocholithiasis could not be established [1, 24, 29].

Other factors were ultimately not independently associated with STC in the present study, such as previous abdominal surgery and upper abdominal surgery. This is despite the higher complication rate and higher risk of conversion in LC in these patients, most likely pertaining to the increased upper abdominal adhesions that can compromise safe peritoneal entry. Perhaps the difficulty of cholecystohepatic triangle dissection is not affected in these patients and therefore the risk of STC is unchanged [30, 31]. There was also no association with increasing BMI, despite the difficulties that can be encountered with entry to the peritoneum and both retraction of the bulky fatty-liver and omentum. Our findings are consistent with Nassar et al. who find no relationship between obesity and operative difficulty according to the Nassar difficulty grade [32–34].

To the best of our knowledge this is the first study to attempt to predict the risk of STC. It offers a simple tool to assess the risk of STC by incorporating high-risk features. It stratifies patients into low, medium and high-risk groups to help counsel surgeons and patients on the risk of STC: in patients with less than 4 features (low and medium risk) the risk of STC is less than 5%; whereas those with 4 or more high-risk features have a risk of at least 19.5%. In those who are high-risk surgical candidates or those who are deemed unfit to tolerate serious complications following STC, patients with a high-risk score may best be managed with conservative measures. Stark differences in rates of intra-operative drain placement, complication, post-operative intervention, prolonged LoS and readmission between STC and CC also emphasise the need for rigorous pre-operative assessment and patient counselling to avoid ill-informed consent [35, 36].

Previous studies have identified risk factors for conversion to open, some of which have also attempted to model the risk of conversion [37–42]. However, it is reported that rates of conversion to open are falling in response to certain laparoscopic salvage alternatives such as STC. In the present study only 0.9% of patients had a conversion to open without STC which calls into question the use of predicting conversion. Whilst conversion to open increases the risk of mortality, hernia, wound infection and both cardiac and respiratory complications, STC too brings its own risks [43, 44]. It is anticipated that the prediction of STC would be more worthwhile and more relevant to patients in contemporary practice [4, 5].

In cases of STC the surgeon should consider whether to continue with the laparoscopic approach and decide if conversion would be worthwhile to prevent the risk of STC. In the STC cohort 8 cases of conversion were performed but did not prevent a STC as the outcome. Aside from the difficulty encountered with the gallbladder dissection itself, conversion to open was of value in three cases: to repair a colonic injury, a duodenal injury and to improve access to the gallbladder itself. It must be highlighted that of the patients that underwent a conventional cholecystectomy, seven of the 23 cases of conversion were performed for significant cholecystohepatic triangle adhesions and did not require STC. Therefore conversion to open may have a role in preventing STC, aside from the management of intra-operative complications and difficulty accessing the gallbladder, but this decision must be made on a case-by-case basis. The operating surgeon must judge the specific benefit of conversion, the likelihood of achieving a conventional cholecystectomy and the patient’s overall fitness for conversion.

In conclusion, the present study incorporates mainly fenestrated STC and illustrates the concerning peri-operative course that follows this procedure. It determines the morbidity of STC and identifies high-risk features associated with STC. A risk model for STC is derived and internally validated to help surgeons identify high-risk patients and both improve pre-operative decision-making and patient counselling.

## Data Availability

Data will be available on request.
